# Predicted Shelf-Life, Thermodynamic Study and Antioxidant Capacity of Breadsticks Fortified with Grape Pomace Powders

**DOI:** 10.3390/foods10112815

**Published:** 2021-11-16

**Authors:** Federico Bianchi, Elisabetta Lomuscio, Corrado Rizzi, Barbara Simonato

**Affiliations:** Department of Biotechnology, University of Verona, Strada Le Grazie 15, 37134 Verona, Italy; federico.bianchi_02@univr.it (F.B.); elisabetta.lomuscio@univr.it (E.L.); barbara.simonato@univr.it (B.S.)

**Keywords:** breadsticks, fortification, grape pomace, OXITEST, shelf-life, thermodynamic study, antioxidant capacity, storage

## Abstract

Grape pomace (GP), is the main winemaking by-product and could represent a valuable functional food ingredient being a source of bioactive compounds, like polyphenols. Polyphenols prevent many non-communicable diseases and could contrast the oxidation reaction in foods. However, the high content in polyunsaturated fatty acid, the described pro-oxidant potential action of some polyphenols and the complex interactions with other components of matrices during food processing must be considered. Indeed, all these factors could promote oxidative reactions and require focused and specific assay. The aims of this study were to evaluate the effects of GP powder (GPP) addition (at 0%, 5% and 10% concentrations) in breadsticks formulations both on the antioxidant activity at room temperature during storage and on the shelf-life by the OXITEST predictive approach. GPP fortification increased the total polyphenols content and the antioxidant activities of breadsticks. FRAP reduced during the first two days of storage at room temperature, TPC increased during the 75 days, while ABTS showed a slight progressive decrease. However, GP negatively influenced OXITEST estimated shelf-life of breadsticks, incrementing the oxidation rate. In conclusion, even if GP fortification of breadsticks could improve the nutritional value of the products, the increased commercial perishability represents a drawback that must be considered.

## 1. Introduction

FAO estimated that food industry annually generates 1.3 billion tons of waste [1], and reducing food wastes and food losses is one of the aims of the 2030 Agenda for Sustainable Development [2]. An alternative purpose for food by-products valorization is their incorporation in different food matrices, thus becoming functional food ingredients and sources of bioactive compounds, such as phenolic compounds, proteins, and dietary fiber (DF) [3,4]. The wine industry generates a considerable amount of waste mainly constituted by grape pomace (GP). GP is a source of bioactive compounds, like polyphenols, minerals and fiber. Therefore, GP could be transformed into a high added-value ingredient, that could improve the nutritional profile of the different food matrices [3,5,6].

Bakery products are consumed worldwide, and they could represent a potential carrier for the delivery of functional ingredients [7,8]. For instance, breadstick is a cylindrical shape of bread, commonly eaten due to its practicality, taste, and crunchiness. Moreover, it’s usually consumed before the meal as an appetizer [9].

As it is known, unsaturated fats, such as olive oil, which represent a common ingredient in breadsticks production, are prone to oxidation [10]. The rancidification is the major cause of oxidative quality losses for fats in foods during their storage. This process leads to off-flavor and harmful to health compounds development. Shelf-life tests based on chemical analyses, during long-term storage provides accurate results, but these approaches are time-consuming, take space and consume many samples [11]. The oven test (or Schaal’s test) is an accelerated shelf-life analyses performed at 60 °C. However, this could take several days to weeks [12]. To save time, the “Oxygen Bomb Method” could be adopted. 

OXITEST operates by accelerated conditions of temperature (room −110 °C) and oxygen or air pressure (0–8 bar). The instrument renders the measure of the absolute pressure change inside two independents, closed and thermostated rooms (A-B), monitoring the oxygen uptake by reactive substrates. Inside the rooms are introduced the titanium plates containing the sample and, when required, one or more spacers. An external cover, provided with screws and a discharged tap, permits the reactor’s hermetic closure, allowing the operator to eliminate the residual atmosphere from the room. The OXISoft™ program included in the instrument calculates the IP value from oxidation curve by a graphical (two tangent) method [13]. At the end of the oxidation test, it is possible to obtain a Test Report including the sample IP and the analysis details for each sample. For estimating the shelf-life, the tested product is analyzed at least at three different temperatures (obtaining three different IP) and at the end, the OXISoft™ program calculate the linear regression equation that allows to extrapolate the IP at room temperature (20–25 °C). Besides the short time required for the assay, the OXITEST can test the oxidative stability of both raw materials and finished products without the fat/oil extraction before analysis required by other approach like Rancimat. OXITEST determines the IP value which correlates with the onset of autooxidation and thus, does not indicate the production of secondary oxidation compounds as in Rancimat [11]. In addition, OXITEST can be used for a wide range of samples, both in liquid and solid form, including meat, oils, mayonnaise, and baked goods with the unique limit that the fat content must be at least 2–4% fat content [13,14,15,16,17,18,19].

The replacement of wheat flour with GP powder (GPP) could impact on oxidative stability. Indeed, since GP contains polyphenols and polyunsaturated fatty acids, it could both inhibit or promote lipid oxidation. Antioxidants can suppress the initiation phase and stop the propagation phase of the oxidation process by reducing the availability of catalyst metals or sequestrating free radicals from the system [20]. 

The present study aimed to evaluate the effects of GPP addition on the shelf-life, rate of reaction and thermodynamic parameters of breadstick oxidation performed with OXITEST and antioxidant activities of products during storage at room temperature. 

## 2. Materials and Methods

### 2.1. Preparation of Grape Pomace Powders 

Grape by-products (*Vitis vinifera* cv. Cabernet), collected after red winemaking, was kindly provided by Ripa Della Volta (Verona, Italy). GP was stabilized as described by Tolve et al. (2020) [21]. Briefly, after GP vacuum oven drying (VD 115 Binder GmbH, Tuttlingen, Germany; 30 kPa, 40 °C) and a final moisture content of 11.0 g water/100 g dry matter, stems and seeds were manually removed. The outcome was milled and sieved under a particle size of 200 μm. The ground pomace obtained (GPP) was stored at room temperature under-vacuum in a dark bag until utilization. 

### 2.2. Preparation of Breadsticks

Breadsticks were kindly produced by Panificio Zorzi (Brentino Belluno, Verona, Italy) [22]. The chemical composition of wheat flour (reported in the label) was carbohydrates 69.9 g/100 g, protein 11.5 g/100 g, fat 1.2 g/100 g and dietary fiber 2 g/100 g. For the preparation of the doughs 8 kg of flour, 0.38 kg of extra virgin olive oil, 200 g of *Saccharomyces cerevisiae*, 150 g of salt and 4.5 L of water have been mixed obtaining the control samples B0. The experimental samples were prepared, replacing wheat flour with 5 and 10 g/100 g of GPP, obtaining (B5 and B10 samples, respectively. Then, doughs have been processed with a professional planetary kneading machine (Planetary Kneading, Sammic, Bergamo, Italy) until a homogeneous dough is obtained. The doughs were pressed with an automatic sheeter (Industrial breadstick machine, Prim s.r.l., Milano, Italy) to obtain breadsticks. After leavening (30 min, 32 °C), breadsticks were cooked at 168 ± 2 °C for 27 min and cooled until room temperature. Finally, 50 g of breadsticks were packaged in a transparent polypropylene film. The water activity of all samples was in the range of 0.185–0.192 ± 0.010 (standard deviation) and did not change during storage. The storage conditions were: 25 °C, in the dark. Preservatives were not added in the formulation and the producer suggested a shelf-life of about 50 days for breadsticks.

### 2.3. Accelerated Oxidation Test of Breadsticks with OXITEST

The OXITEST apparatus (Velp^®^ Scientifica, Usmate (MI), Italy) is constituted by two titanium chambers, both thermostated and hermetically sealed. Each chamber (A and B, respectively) can contain up to three titanium sample holders [17]. In our study, all holders have been required for samples, and titanium spacers (used to keep constant the void volume in each chamber) were not used. Fifty grams of breadsticks (one package) were ground (Moulinex, Bagnolet, France) and loaded in each reactor chamber. The accelerated oxidation test was repeated twice for a total of four replies. The OXITEST response is the induction period (IP), expressed as “stability time” before fat oxidation and corresponded to the drop of O_2_ pressure due to its consumption by the sample, and it is estimated graphically (two tangent method). The accelerated shelf-life test was performed at three different temperatures (90, 100, and 110 °C) under 0.6 MPa pressure of pure oxygen and data were collected every one minute. The range of temperature were in accord to OXITEST manufacturer and sample type. Caruso et al. (2017) used similar range of temperature (80–110 °C) for bakery products [13]. Moreover, other authors have validated the OXITEST method against the Rancimat one operating in this range of temperature. The data were analyzed using the OXISoft™ program (Velp^®^ Scientifica, Usmate (MI), Italy). With this procedure, it is possible to estimate the shelf-life of samples at room temperature, in the range of 20–25 °C [11,13,16].

#### Study of Oxidation Kinetic

Pressure data obtained from the OXITEST were transformed in moles of oxygen using the ideal gas law (1):

(1) $$n=\frac{PV}{RT}$$

where *n* is the mole of oxygen, *P* is the pressure (bar), *V* is the remaining volume of the gas in the chamber after putting the sample and the plate (m^3^), *R* is the constant gas law, and *T* is the temperature (K). Then, we set to pseudo-first-order oxidation kinetics to estimate the oxidation rate constant (k), plotting the mole of oxygen value by time (2):

(2) $$C=C_{0}\cdot \;e^{-kt}$$

where *C*_0_ represents the initial mole of oxygen value in the reactor of the OXITEST device, k introduces the rate constant for oxidation kinetics (1/day), *C* represents the oxygen mole that varies with time, and time is defined as *t* in days.

### 2.4. Thermodynamic Study

The activation energy of the reactions was obtained using the Arrhenius equation in logarithm form for the three temperatures studied (3):

(3) $$\ln\left(k\right)=\ln\left(A\right)-\frac{E_{a}}{RT}$$

where *A* is the pre-exponential factor, *Ea* is the activation energy (kJ/mol), *R* is the molar gas constant (8.314510 J/K mol), and *T* is the absolute temperature in Kelvin.

Enthalpy (Δ*H*) and entropy (Δ*S*) of the activated states were determined by the regression of ln (k/T) vs. 1/T, equation derived from the theory of the activated complex and expressed by Eyring Equation (4):

(4) $$\ln\left(\frac{k}{T}\right)=\frac{-\Delta H}{R}\cdot \;\frac{1}{T}+\left\{\ln\left(\frac{k_{b}}{h}\right)+\frac{\Delta S}{R}\right\}$$

where “*k_b_*” is the Boltzmann’s constant, “*h*” is the Plank constant, and “*T*” is the absolute temperature (K). Δ*H* represents the enthalpy of activation, and Δ*S* the entropy of activation. The Gibbs free energy was obtained from the relation between Δ*H*, Δ*S*, and temperature *T* (5):

(5) ΔG=ΔH−ΔS·T

### 2.5. Total Phenol Content, Antioxidant Activities FRAP and ABTS

Every 15 days, starting from the day of breadsticks production (T0), breadstick from one pack for each formulation were minced. The study lasted for 75 days (T5) according to the shelf-life of breadsticks determined with the OXITEST. Grounded breadsticks were utilized for phenolic extraction by stirring 0.5 g of sample with 7.5 mL of MeOH:HCl 97:3 (*v*/*v*) for 16 h in the dark at room temperature. Supernatants were collected after centrifugation (3500× *g* for 10 min) and used for Folin-Ciocalteu total phenolic compounds (TPC), ABTS (2,20-azino-bis (3-ethylbenzothiazoline-6-sulfonic acid)) and FRAP (ferric reducing ability of plasma) radical scavenging activities determination. TPC, FRAP and ABTS determination were performed as described by Tolve et al. (2021) [23].

### 2.6. Statistical Analysis

All data were reported as mean values ± standard deviation of at least three measurements. The analysis of variance (two-way ANOVA) with a post hoc Tukey test at *p* < 0.05, has been used for mean comparison. Statistical analyses were performed using the software XLSTAT Premium Version (2021.1.1, Addinsoft SARL, Paris, France).

## 3. Results and Discussion

### 3.1. Accelerated Oxidation Test with OXITEST and Kinetic Study

Table 1 report the induction period (IP) of oxidation for each breadstick’s formulation. Both the amount of GPP addition and temperatures affected the onset oxidation of breadsticks. B0 had higher IP at all temperatures compared to B5 and B10, and similarly B5 to B10. Caruso et al. (2017) reported a IP of 39 h (almost 1.5 times greater than B0) for breadsticks with olive oil [13]. These differences could rely on the different types of olive oil and the manufacturing process of breadsticks used in the current study. Moreover, the increasing temperature in the oxidation chamber led to a significant drop of IP values, indicating an acceleration of the oxidation process as expected and confirmed by the rate of oxidation “k” (Table 1). Indeed, B10 showed a faster rate of oxidation for all tested temperatures compared to B5 and B0.

OXISoft™ calculated the linear regression between the logarithm of the IP and the temperature (°C) to estimate shelf-life (IP in days) at 25 and 20 °C (Table 2). All linear regressions had a coefficient of determination (R^2^) greater than 0.98. Results indicated that B0 had the most extended estimated shelf-life while B10 accounted for the lowest. Under this point of view, B0 had almost two-fold shelf-life days compared to B10.

### 3.2. Thermodynamic Study

The energy of activation *E_a_* represents the minimum amount of energy required for a chemical reaction. In the current study, the *E_a_* ranged from 78.22 to 56.99 kJ/mol (Table 3). The reduced amount of energy for sample B10 compared to B0 and B5 indicated the promotion of oxidative process; indeed, B10 had the lowest shelf-life in this study.

A reaction could be spontaneous or non-spontaneous, exergonic, or endergonic, and exothermic or endothermic. A positive value of enthalpy indicates that the process is endothermic. This finding was also observed during the oxidation of vegetable oil [24]. The negative value of entropy along with positive enthalpy is the sign of non-spontaneous reaction [25]. Moreover, the negative entropy might be the result of associated mechanisms [25,26,27]. As expected, the Gibbs free energy variations for the oxidation (e.g., for samples at 90 °C) were found to be positive, which underlined the non-spontaneous and endergonic nature of the oxidation reaction of breadsticks.

### 3.3. Total Phenol Content (TPC) and Antioxidant Activities (FRAP and ABTS) in Fortified Breadsticks at Room Temperature

**Figure 1:**
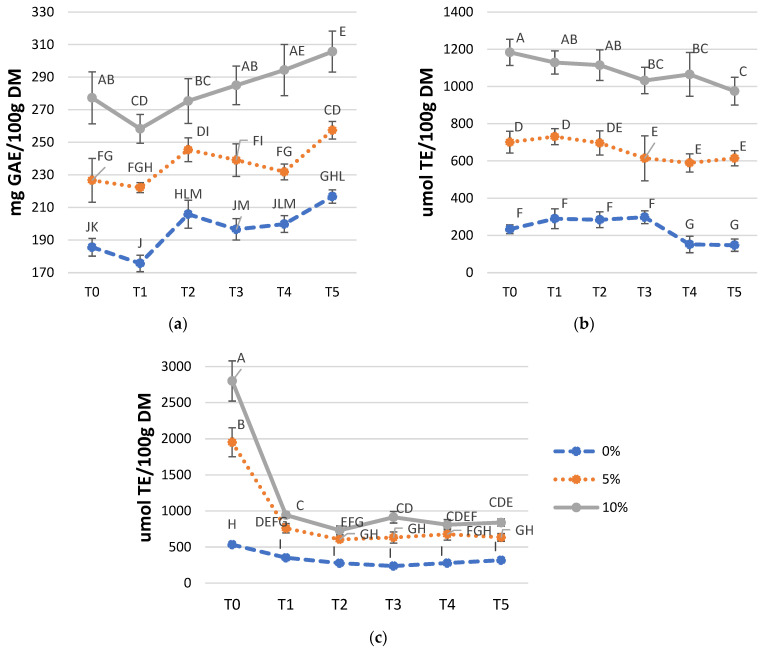
To different letters correspond different means within the same graph (*p* < 0.05). Gray line stands for B10, orange line for B5, and blue line for B0; (**a**) TPC; (**b**) ABTS; (**c**) FRAP. GAE: gallic acid equivalent; TE: Trolox equivalent; DM: dry matter.

Curves of TPC, FRAP, ABTS at different days of sampling are reported in Figure 1. Generally, tested TPC and antioxidant assays had significant differences among samples B0, B5, and B10 (*p* < 0.05). In terms of TPC (panel a, Figure 1), B10 had the highest values compared to B5 and B0. The first two times of storage, TPC decreased even if it was not statistically significant. While from T2 to T5 all samples showed an apparent progressive increase of TPC. Cisneros-Yupanqui et al. (2020) hypothesized that the GP TPC initial reduction during storage resulted from an inhibition of the protein glycation and the trapping of dicarbonyls performed by phenols [28], as also described by Mazumder et al. (2019) [29]. In addition, the progressive increase in TPC in stored GP was attributed to the residual activity of some enzymes of GP powder that could enhance the polyphenols release during the storage [30]. Besides the persistence of enzymatic activities of GP in breadstick, it is possible to hypothesize a rearrangement of other components of the matrix (such as proteins and starch) that can cause a progressive release of previously bound or trapped phenols.

In fortified samples, ABTS increased proportionally to the addition of GPP, independently from the day of storage (panel b, Figure 1). From T0 to T5, there is no evident trend, but antioxidant capacity within T5 was statistically lower compared to T0, for all samples. This indicates that breadsticks’ oxidation started, lowering antioxidant activities, and these results agreed with OXITEST predicted shelf-life.

FRAP values were statistically different between samples (panel c, Figure 1). For example, B5 and B10 had statistically higher antioxidant capacity at the beginning of the study (T0); however, a reduction occurred in 15 days (T1). At the same time, the reduction of FRAP antioxidant capacity was slightly lower for B0. In the current study, the method for extracting the antioxidant molecules is optimized for phenols, while other components could play a role in the different antioxidant activities. For example, Maillard reaction products generated during the thermal processing could be considered responsible for enhancing the antioxidant activities, as mentioned by Chamorro et al. (2012) [31]. Therefore, the two antioxidant activities tests can differently quantify these substances.

Apparently, in the current study, GPP polyphenols did not exert antioxidant activities against breadsticks lipid oxidations and did not prolong the shelf-life of products. This could be explained considering different factors. At first, the addition of GPP could have increased the polyunsaturated fatty acid (PUFA) concentration [32,33] in breadstick, and this could result in an earlier oxidation onset [34]. Moreover, Cabernet cultivar grape pomace is described to have about 9 g/100 g DM (Dry Matter) of fat, and more than 50% are PUFA [35]. Under this point of view, the dramatic decrease in antioxidant power of GP measured by FRAP could suggest the quick consumption of antioxidant molecules present in GP that are detectable only at T0. Finally, a pro-oxidant action of GP component should be evaluated [36].

## 4. Conclusions

The fortification of breadsticks with GPP negatively affected the OXITEST predicted shelf-life of samples B5 and B10. The rate of oxidation increased accordingly to the temperature, especially in B10. The thermodynamic study underlined that the oxidation process is non-spontaneous, endergonic, and endothermic. We monitored breadstick TPC, ABTS and FRAP for 75 days according to OXITEST predicted shelf-life at room temperature. TPC was slightly affected during the time span, and all samples had higher TPC at the end of the analysis, while ABTS showed a slight decrease and FRAP showed a substantial decrease within the first two days of sampling. B10 always had a higher antioxidant capacity and phenol content compared to B5 and B10. In conclusion, GPP represented an interesting environmentally friendly ingredient for food fortification, which could enhance the antioxidant capacity of food matrixes. However, in terms of fat oxidation in food, GPP had a shortening effect on breadstick shelf-life, probably due to pro-oxidant molecules or the presence of polyunsaturated fatty acids.

OXITEST is a novel and innovative technique to predict shelf-life of fortified breadsticks. Anyway, the method would require a validation with other consolidate tests, such as Schaal’s test. Indeed, high temperature and pressure could promote unexpected interactions among different molecules in foods, generating misrepresented shelf-life prediction. Moreover, secondary products of oxidation need to be evaluated with different methods, since they are responsible of off-flavors and toxicity of rancidification. For these reasons, further studies are required to assess the real impact of GPP on fat-containing bakery products shelf-life.

## Figures and Tables

**Table 1:** Induction period (IP) and rate of oxidation *k* of control and fortified with grape pomace (GP) breadsticks. OXITEST was set at temperatures 90, 100, and 110 °C.

Samples	IP (min)			Rate of Oxidation *k* (1/Day)		
	90 °C	100 °C	110 °C	90 °C	100 °C	110 °C
B0	1465.5 ± 48.8 ^a^	747.5 ± 29.0 ^b^	419.5 ± 7.8 ^f^	0.2053 ± 0.0012 ^a^	0.4132 ± 0.0025 ^d^	0.7710 ± 0.0047 ^g^
B5	776.5 ± 44.5 ^b^	386.5 ± 6.4 ^d^	218 ± 4.2 ^g^	0.2307 ± 0.0015 ^b^	0.4890 ± 0.0027 ^e^	0.8201 ± 0.0052 ^h^
B10	698.5 ± 9.2 ^c^	331.5 ± 7.8 ^e^	201 ± 8.5 ^h^	0.3010 ± 0.0020 ^c^	0.5185 ± 0.0032 ^f^	0.9295 ± 0.0064 ^i^

Values with different superscript letters are statistically different for *p* < 0.05. B0: control breadstick; B5: breadsticks fortified with 5% of GP; B10: breadsticks fortified with 10% of GP.

**Table 2:** Estimation of shelf-life of control and fortified breadsticks at 20 and 25 °C. The coefficient of determination (R^2^) of linear regression performed by OXISoft™ program is also reported.

Samples	R^2^	Shelf-Life 25 °C (days)	Shelf-Life 20 °C (days)
B0	0.9981	58	80
B5	0.9967	33	45
B10	0.9870	27	36

B0: control breadsticks; B5: breadsticks fortified with 5% of GP; B10: breadsticks fortified with 10% of GP.

**Table 3:** Energy of activation (*E_a_*), enthalpy of activation (Δ*H*), entropy of activation (Δ*S*), and determination of Gibbs free energy at 90 °C (Δ*G* 90 °C) of control and fortified samples (B0, B5, and B10).

Samples	*E_a_* (kJ/mol)	Δ*H* (kJ/mol)	Δ*S* (J/K mol)	Δ*G* 90 °C (kJ/mol)
B0	77.53 ± 2.65 ^a^	74.43 ± 2.65 ^a^	−127.36 ± 7.20 ^a^	120.68 ± 1.04 ^a^
B5	78.22 ± 0.96 ^a^	75.12 ± 0.96 ^a^	−122.45 ± 2.73 ^a^	119.58 ± 1.95 ^a^
B10	56.99 ± 1.52 ^b^	53.89 ± 1.52 ^b^	−177.36 ± 3.84 ^b^	118.30 ± 2.91 ^a^

Values with different superscript letters are statistically different for *p* < 0.05. B0: control breadsticks; B5: breadsticks fortified with 5% of GP; B10: breadsticks fortified with 10% of GP.

## Data Availability

Not applicable.
